# A random six-phase switch regulates pneumococcal virulence via global epigenetic changes

**DOI:** 10.1038/ncomms6055

**Published:** 2014-09-30

**Authors:** Ana Sousa Manso, Melissa H. Chai, John M. Atack, Leonardo Furi, Megan De Ste Croix, Richard Haigh, Claudia Trappetti, Abiodun D. Ogunniyi, Lucy K. Shewell, Matthew Boitano, Tyson A. Clark, Jonas Korlach, Matthew Blades, Evgeny Mirkes, Alexander N. Gorban, James C. Paton, Michael P. Jennings, Marco R. Oggioni

**Affiliations:** 1 Department of Genetics, University of Leicester, Leicester LE1 7RH, UK; 2 Dipartimento di Biotechnologie Mediche, Università di Siena, 53100 Siena, Italy; 3 Research Centre for Infectious Diseases, School of Molecular and Biomedical Science, University of Adelaide, Adelaide, South Australia 5005, Australia; 4 Institute for Glycomics, Griffith University, Southport, Queensland 4215, Australia; 5 Pacific Biosciences, Menlo Park, California 94025, USA; 6 Bioinformatics and Biostatistics Analysis Support Hub, University of Leicester, Leicester LE1 7RH, UK; 7 Department of Mathematics, University of Leicester, Leicester LE1 7RH, UK; 8 These authors contributed equally to this work

## Abstract

*Streptococcus pneumoniae* (the pneumococcus) is the world’s foremost bacterial pathogen in both morbidity and mortality. Switching between phenotypic forms (or ‘phases’) that favour asymptomatic carriage or invasive disease was first reported in 1933. Here, we show that the underlying mechanism for such phase variation consists of genetic rearrangements in a Type I restriction-modification system (SpnD39III). The rearrangements generate six alternative specificities with distinct methylation patterns, as defined by single-molecule, real-time (SMRT) methylomics. The SpnD39III variants have distinct gene expression profiles. We demonstrate distinct virulence in experimental infection and *in vivo* selection for switching between SpnD39III variants. SpnD39III is ubiquitous in pneumococci, indicating an essential role in its biology. Future studies must recognize the potential for switching between these heretofore undetectable, differentiated pneumococcal subpopulations *in vitro* and *in vivo*. Similar systems exist in other bacterial genera, indicating the potential for broad exploitation of epigenetic gene regulation.

## Abstract (web summary)

Pneumococci can alternate between harmless and highly virulent forms. Here the authors show that such variation may be due to random rearrangements in a genetic locus encoding a restriction-modification system, resulting in epigenetic changes that affect expression of many genes.

## Graphical abstract

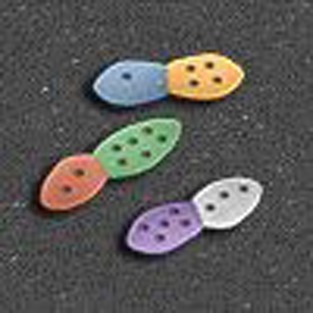

The importance of *Streptococcus pneumoniae* as a human pathogen has prompted intense investigation in the post genomics era, with more genome sequences available than for most other species^1^. In spite of this, much remains to be elucidated regarding the molecular basis of critical virulence attributes, including the phenomenon of opacity phase variation (high frequency, reversible switching of expression)^2^,^3^. Examination of multiple genomes has failed to identify nucleotide polymorphisms or accessory regions that could be consistently associated with virulence phenotypes; however, such studies have ignored the potential of restriction-modification (RM) systems to mediate gene regulation via epigenetic changes^4^,^5^. The *S. pneumoniae* genome contains two Type I, three Type II and one Type IV RM systems^6^,^7^; of these, only the DpnI Type II RM system has been described in detail in the past^8^. One of the Type I RM systems, which we propose to name SpnD39III according to REBASE criteria, contains three co-transcribed genes: *hsdR*, *hsdM* and *hsdS* (coding for SpnD39III, M.SpnD39III and S3.SpnD39III); there is also a separately transcribed Cre tryrosine DNA recombinase gene and two truncated *hsdS* genes (S1.SpnD39III and S2.SpnD39III) downstream^5^,^9^. The actively transcribed *hsdS* gene contains two variable regions that encode the two target recognition domains (TRD) of S.SpnD39III, and it shares inverted repeats with the truncated *hsdS* genes; these truncated genes encode additional, separate alleles for both TRDs, but lack consensus *hsdS* 5′ ends, ribosomal binding sites and promoters. The series of inverted repeat regions in the *hsdS* genes had been shown to enable recombination, thought to be facilitated by the CreX recombinase, potentially generating alternative *hsdS* variants encoding enzymes with different target specificities^6^,^10^. Similar phase variable Type I RM systems have been described in *Bacteroides fragilis* and *Mycoplasma pulmonis*^11^,^12^.

Here, we show that recombination between *hsdS* genes confers six different target specificities to the Type I SpnD39III RM system and that this has significant impact on gene expression and virulence of pneumococci.

## Results

### Characterization of the SpnD39III RM system

**Figure 1:**
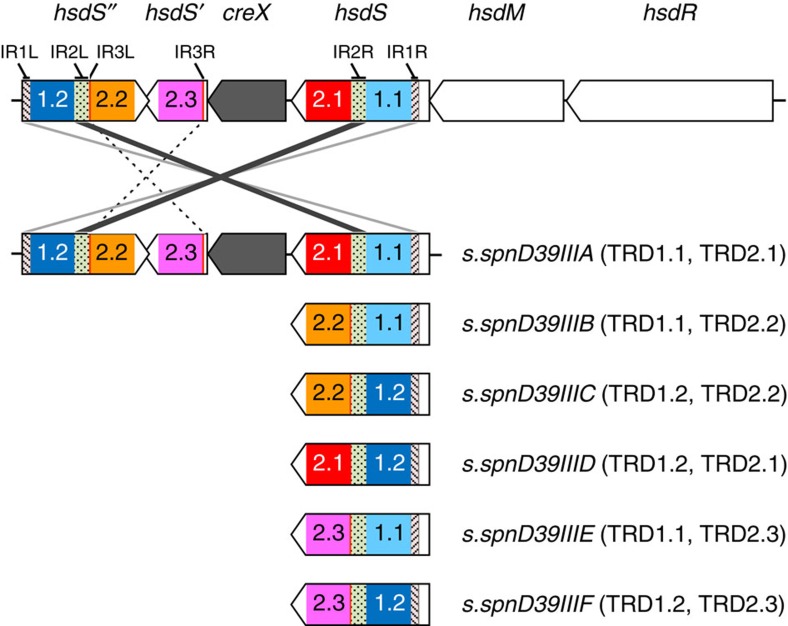
Schematic map of the SpnD39III locus and of the six alternative *hsdS* genes. In strain D39, the Type I RM system SpnD39III locus (upper row from right to left) includes *hsdR* (locus_tag SPD_0455; GenBank protein database accession code ABJ53942; REBASE SpnD39ORF782P), *hsdM* (SPD_0454; ABJ54850; M.SpnD39ORF454P) and *hsdS* (SPD_0453; ABJ53819; S3.SpnD39ORF454P), a Cre recombinase (SPD_0452; ABJ54057), a truncated *hsdS′* gene with only one variable domain (SPD_0450; ABJ55250; S2.SpnD39ORF454P) and a further truncated *hsdS′′* gene with two variable domains (SPD_0451; ABJ54176; S1.SpnD39ORF454P). Three series of inverted repeats (IR1 to IR3) allow for recombination. These rearrangements, exemplified by crosses between the IRs, allow formation of six different variant *hsdS* alleles encoding different HsdS proteins SpnD39IIIA-F. Inverted repeats are of 85 (IR1, striped), 333 (IR2, dotted) and 15 bp (IR3). The six recombinant *s.spnD39IIIA-F* alleles (Genbank nucleotide database accession codes KJ955483, KJ955484, KJ955485, KJ955486, KJ398403 and KJ398404) are shown below with their TRDs numbered according to Table 1.

To characterize the SpnD39III RM enzyme, we constructed mutants in the virulent pneumococcal strain D39 each expressing only one of the six possible *hsdS* variants (Fig. 1, Table 1). The strains are designated SpnD39IIIA-F and are characterized by a single S.SpnD39IIIA-F variant, respectively, referred to as ‘locked’ strains (Fig. 1, Table 1). Mutants were constructed by deleting the truncated *hsdS genes* downstream of the actively transcribed locus and selecting at least one strain with a ‘locked’ *s.spnD39III* allele for each of the six possible variants. Absence of any other mutation was confirmed by whole-genome sequencing. Single-molecule real-time (SMRT) sequencing and methylome analysis^13^ identified distinct N6-adenine methylation targets for each SpnD39IIIA-E variant (Table 1, Supplementary Fig. 1). Methylome data of the locked strains showed methylation in both strands of over 99% of the target sites (Table 1, Supplementary Fig. 1). On the basis of these results, each target recognition domain can be assigned to a specific sequence that it is responsible for recognizing: the amino-terminal (N-terminal) TRDs of SpnD39IIIA, IIIB and IIIE (TRD1.1) recognize CRAA; the N-terminal TRDs of IIIC and IIID (TRD1.2) recognize CAC; the carboxyl-terminal (C-terminal) TRDs of IIIA and IIID (TRD2.1) recognize CTG; the C-terminal TRDs of IIIB and IIIC (TRD2.2) recognize TTC; and the C-terminal TRDs of IIIE (TRD2.3) recognize CTT (Table 1, Fig. 1). Given the target specificities identified for SpnD39IIIA-E, we could infer the target specificity also for SpnD39IIIFP, where the P stands for putative^10^. Thus, by recombination between these five different TRDs (TRD1.1 or 1.2 and TRD2.1, 2.2 or 2.3), six different methylation specificities are possible. Three of these bipartite target sites, a distinct feature of Type I enzymes, were experimentally confirmed with restriction inhibition assays using plasmid DNA isolated from D39 strains containing locked S.SpnD39IIIA, B or C enzymes (Supplementary Fig. 2a–c). The SMRT methylome analysis of *S. pneumoniae* D39 also identified two active Type II RM systems (SpnD39I—TCTAG[^m6^A]; and SpnD39II—TCG[^m6^A]C). D39 contains a second Type I system which is non-functional through truncation of the SpnD39ORF782P gene (Table 1). However, methylome analysis of *S. pneumoniae* clinical isolates WCH16 and WCH43 (ref. 14) has allowed us to identify the equivalent Type I RM system with two allelic variants of which also showing a potentially phase variable methylase. This system contains variants A and B that were found to methylate the sequences G(^m6^A)YN_6_TATC and TG(^m6^A)N_7_TATC, respectively (Table 1). Examination of all available bacterial genome sequences reveals similar arrangements of *hsdS* genes in other genera, thus potentially allowing for similar reversible switching in other species (Supplementary Table 1)^11^,^12^.

### SpnD39III target site distribution

Natural switching between the six distinct S.SpnD39III variants resulted in different numbers, positions and types of methylation sites in the D39 genome; they ranged in number from 424 sites (SpnD39IIID) to 1,029 sites (SpnD39IIIB). The expected numbers of sites, based on the nucleotide base occurrence in the D39 genome, differed significantly from those observed, with SpnD39IIIA, SpnD39IIIB and SpnD39IIIE showing 50% more sites than predicted, and SpnD39IIIC, SpnD39IIID and SpnD39IIIF being underrepresented (Table 1). Being non-palindromic, the SpnD39III sites could be mapped to one of the two strands of the genome, where the localization to one strand does not refer to the position of the site but rather to the orientation of site and RM enzyme complex. Intriguingly, we observed not only a marked preference for all SpnD39III variants to be on the lagging strand (Pearson’s *χ*^2^-test, *P*<0.001) (Supplementary Fig. 3), but also that the deviation seen between expected and observed sites mapped only to one strand of the genome, that is, overrepresented sites were overrepresented on the lagging strand and underrepresented sites were underrepresented on the leading strand (Supplementary Table 2). Furthermore, reciprocal positioning of the sites on the leading and lagging strands of the genome showed a non-random pattern for all SpnD39III sites except SpnD39IIIE (Supplementary Tables 3 and 4). These observations were not explained by the GC skew of the genome and were not observed when testing classical representatives of Type I RM families A–C such as EcoK1, EcoAI and EcoR124I (Supplementary Tables 2–4). Interestingly, the target sites of Type III RM systems EcoP1I and EcoP15I (shown to be most active in the presence of non-co-directional, head-to-head oriented target sites)^15^ also showed significant non-random distribution between strands in the genome, when tested either by nearest neighbour distribution using the Clark–Evans test, or by target site pair distribution using the Kolmogorov–Smirnov test (Supplementary Tables 2–4). Although these calculations did not allow us to draw direct conclusions on functionality, they indicated that the SpnD39III site distribution pattern had more in common with Type III RM systems, which have directional activity, than with the classical Type I systems. An alternative conclusion could be that the observed genome-wide asymmetric localization of nucleotide signatures is associated with a functional role in replication or transcription^16^. Bioinformatics analysis showed no obvious association of the methylation target sites with promoters, small RNAs, genes or operons. Out of the six variants, SpnD39IIIA, SpnD39IIIB, SpnD39IIID and SpnD39IIIF showed reduced occurrence in non-coding regions (Pearson’s *χ*^2^-test, *P*<0.001).

### Evaluation of SpnD39III methylation and restriction activity

**Figure 2:**
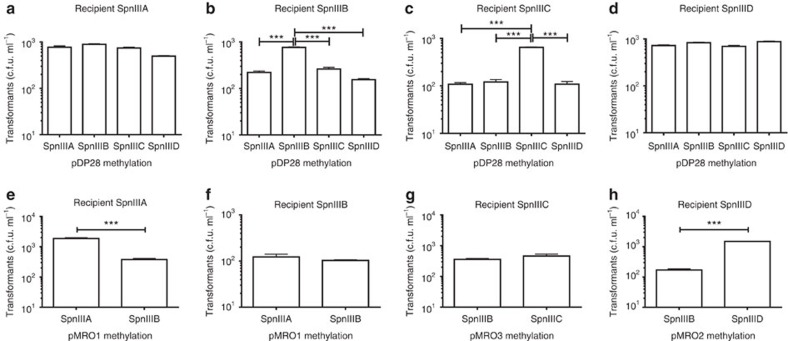
SpnD39III restriction. (**a**–**h**) Restriction by SpnD39III was demonstrated by transformation of differently methylated pDP28 plasmids into strains expressing only one SpnD39III variant. Restriction was evident in strains expressing variant SpnD39IIIB (**b**) and SpnD39IIIC (**c**), which have non-co-directional target sites on pDP28 (Supplementary Fig. 4), whereas restriction was not evident in strains expressing variants SpnD39IIIA (**a**) or SpnD39IIID (**d**), which have co-directional target sites on pDP28. Graphs indicate s.d. of three independent replicates. Statistically significant differences found using an analysis of variance Tukey test are shown. When utilizing recombinant pDP28 derivatives with two non-co-directional target sites for SpnD39IIIA (**e**) or SpnD39IIID (**h**) restriction could also be observed for these sites, whereas no restriction was observed when transforming a recombinant pDP28 with only one SpnD39IIIB site (**f**) or two co-directional SpnD39IIIC sites (**g**). ***P<0.001.

To evaluate the mechanism of restriction by SpnD39III, we extracted differently methylated forms of the shuttle vector pDP28 from the locked SpnD39III strains (Supplementary Fig. 4) and retransformed these plasmids into the different locked SpnD39III strains (equivalent phage infection experiments are currently not possible due to the lack of phage capable of infecting encapsulated pneumococci). Reduced transformation efficiencies, indicating restriction of incoming plasmid DNA, were only observed when transforming heterologously methylated plasmids into the SpnD39IIIB or SpnD39IIIC strains (Fig. 2a–d). Importantly, the SpnD39IIIB and SpnD39IIIC sites of pDP28 are non-co-directional unlike the SpnD39IIIA and SpnD39IIID sites (Supplementary Fig. 4). These findings add further to the novel nature of this system as the importance of non-co-directional positioning of target sites has previously only been reported for Type III RM systems^4^. We hypothesize that this novel observation (directional restriction activity of a Type I RM system) may be related to the peculiarity of our model test system, which is based on the integration of non-methylated single-stranded DNA into a resident rolling circle replicating plasmid during natural transformation (see also the legend of Supplementary Fig. 4). Indeed, no restriction could be observed when transforming linear DNA with differently positioned SpnD39IIIA sites into the chromosome (Supplementary Fig. 2f), an observation that is more in line with what is expected during natural transformation^17^. To further test our findings, we constructed recombinant pDP28 derivatives containing either: (i) non-co-directional SpnD39IIIA or SpnD39IIID sites, (ii) a unique SpnD39IIIB site or (iii) co-directional SpnD39IIIC sites (Supplementary Fig. 4b–d). Transformation of these plasmids confirmed that, in our specific model system at least, two non-co-directional sites are needed for restriction (Fig. 2e–h). It is possible therefore that the preferential co-directional site distribution in the genome (see above) could indicate a selective advantage in reducing risks of self-cleavage following switching between SpnD39III alleles.

### SpnD39III-dependent changes in gene expression

**Figure 3:**
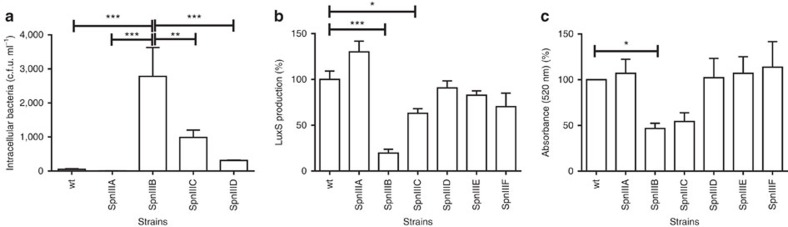
*In vitro* phenotypes of SpnD39IIIA-F variant strains. (**a**) Phagocytosis of pneumococci by RAW 264.7 macrophages. Mean values of three independent replicates and s.d. are shown. (**b**) LuxS expression was assayed by quantitative western blot and the mean of quadruplicate samples are shown (in case of SpnD39E-F including two technical replicates). (**c**) The production of the type 2 polysaccharide capsule was quantified using an uronic acid assay and the mean and s.d. of quadruplicate samples are shown (in case of SpnD39E-F including two technical replicates). Statistical analysis of *in vitro* experiments (**a**–**c**) was performed using one-way analysis of variance and a Tukey post-comparison test. *P<0.05, **P<0.01, ***P<0.001.

To determine the global impact of the altered genomic methylation patterns that result from switching between the alternate SpnD39III specificities, we examined gene expression by RNA-seq in four of the variants (SpnD39IIIA-D). In all locked strains, changes in gene expression could be observed (the RNA-seq data have been deposited in the NCBI GEO database with accession code GSE55182). The most striking change was a downregulation (relative to all other variants) of the capsule operon in SpnD39IIIB. The polysaccharide capsule is a major pneumococcal virulence determinant^18^. In addition, the *dexB*, *luxS* and SPD_310 genes, which are all located close to the capsule operon in the genome, were also downregulated in SpnD39IIIB (Table 2). The reduced production of LuxS and capsular polysaccharide in the SpnD39IIIB strain were confirmed by quantitative Western blot and capsule assay, respectively (Fig. 3b,c). In SpnD39IIIA, the *blpY*-SPD_0475 operon^19^, the sucrose regulator, and the fucose operon were significantly downregulated, and a series of genes, including *psaABC* and *dnaK,* were upregulated relative to the other three variants tested (Table 3). SpnD39IIIC and SpnD39IIID strains did not show significant differential regulation of any other genes with respect to the other variants under these assay conditions. No SpnD39III sites could be identified in the promoter or regulatory regions of any of the differentially regulated genes, so the exact method by which differential methylation affects gene regulation patterns in the pneumococcus remains to be elucidated (Tables 2 and 3). These data do, however, show that genetic recombination at the *spnD39III* locus results in a significant impact on global gene expression patterns via epigenetic mechanisms thereby differentiating the pneumococcus into distinct cellular phenotypes.

### Phenotypic impact of SpnD39III phase variation

**Figure 4:**
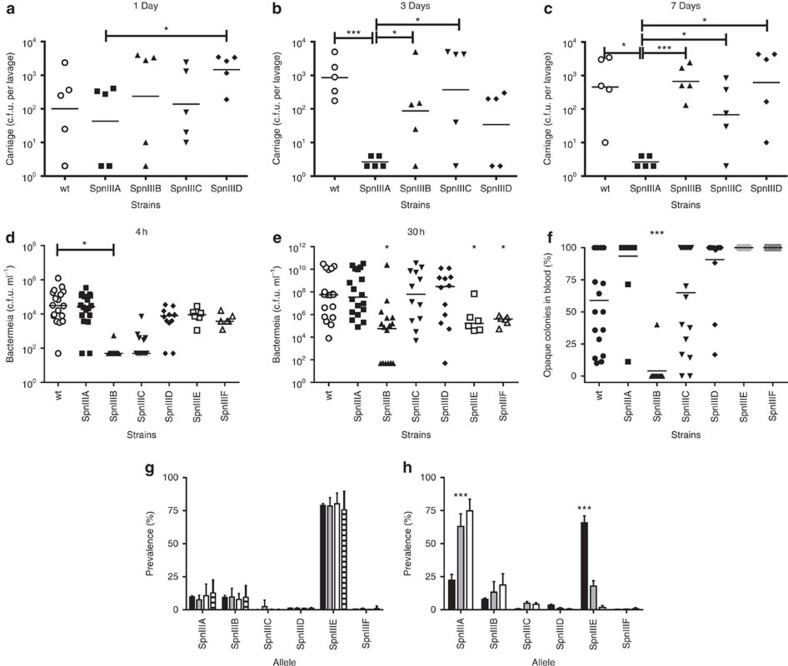
*In vivo* phenotypes of SpnD39IIIA-F variant strains. D39 wt or derivatives expressing only a locked SpnD39IIIA to SpnD39IIIF variant were used in these experiments. Carriage experiments were performed by intranasal inoculation of 5 × 10^4^ pneumococci into BALB/c mice, which are resistant to invasive infection. (**a**–**c**) The extent of carriage was evaluated by nasal lavage at day 1 (**a**), day 3 (**b**) and day 7 (**c**). (**d**,**e**) In the invasive disease model, susceptible CD1 mice received an intravenous challenge of 1 × 10^5^ pneumococci, and blood samples were taken at 4 and 30 h post challenge. Statistical significance of differing bacterial load in D39 wt or SpnD39III variant infected mice was calculated on log-transformed data using the unpaired (two-tailed) *t*-test. (**f**) Blood samples plated directly onto catalase agar plates were examined and the percentage of opaque colonies observed for each SpnD39III variant strain. (**g**,**h**) Allele quantification in D39 wt was performed on DNA extracted from the nasal lavages of the carriage experiment (see also Supplementary Table 5) or on DNA extracted from colonies grown from blood samples in the invasive disease experiment and SpnD39III variant percentages for each are represented respectively in **g** (inoculum black, 1 day grey, 3 days white, and 7 days striped) and **h** (inoculum black, 4 h grey and 30 h white). *P<0.05, **P<0.01, ***P<0.001.

Certain SpnD39III alleles also impacted on colony opacity. This morphological feature is known to undergo reversible phase variation between opaque (OP) and transparent (TP) phenotypes via an unknown mechanism; the OP phenotype is preferentially associated with invasive disease and TP with carriage^3^. The SpnD39III-locked variant strains differed considerably in their opacity: SpnD39IIIA and SpnD39IIIE strains yielded 100% OP colonies; SpnD39IIIB 7% OP colonies; SpnD39IIIC 25% OP colonies; SpnD39IIID 59% OP colonies; and SpnD39IIIF 96% OP colonies (Supplementary Fig. 2d,e). The impact of the distinct SpnD39III variant methylation patterns was therefore investigated in murine models of nasopharyngeal colonization and invasive disease. The locked SpnD39IIIA, unlike the other variants or the D39 wild type, was unable to stably colonize the nasopharynx (Fig. 4a–c; Supplementary Fig. 5). In contrast, during invasive infection, those mice infected with the locked SpnD39IIIB had lower bacterial counts in their blood at both 4 and 30 h after challenge than mice infected with the strains expressing the other SpnD39III variants (Fig. 4d,e). At a later time-point, the locked SpnD39IIIE and SpnD39IIIF strains also showed lower virulence in this model (Fig. 4d,e). The lower virulence of SpnD39IIIB is consistent with the lower capsule expression seen in the gene expression studies (Table 2 and Fig. 3c). Strains expressing SpnD39IIIB also showed an increased susceptibility to phagocytosis by macrophages (Fig. 3a). Animals challenged with SpnD39IIIA yielded almost exclusively OP colonies when bacteria were isolated 30 h post challenge, while animals challenged with SpnD39IIIB yielded mostly TP colonies under the same conditions (Fig. 4f). Thus, differences in opacity can be correlated with specific SpnD39III-locked-allele type; SpnD39IIIA yielded mainly opaque colonies, showed high virulence and therefore poor colonization capacity, whereas SpnD39IIIB yielded mainly transparent colonies and had low systemic virulence. It can therefore be concluded that SpnD39III-mediated epigenetic modification significantly impacts on both opacity phenotype and virulence.

### Quantification of SpnD39III subpopulations

To determine whether genetic switching at the *spnD39III* locus and the consequent differential epigenetic modification was selected for *in vivo*, we analysed the frequencies of the various *spnD39III* alleles in wild-type pneumococci during infection using a wild-type D39 strain in which the SpnD39III locus is free to switch between the six different allele variants. We developed a fluorescent GeneScan assay (fragment length analysis) using PCR followed by restriction digest of the products, specifically for this purpose, which allows the simultaneous identification and quantification of all six alleles within the bacterial population (an example of the methodology, and exemplar results are shown in Supplementary Fig. 6a–c; verification of this method is shown in Supplementary Fig. 6d,e). The D39 inoculum that was used to challenge mice intravenously (Fig. 4d,e) was found to be predominantly *spnD39IIIE* (12% *spnD39IIIA*, 13% *spnD39IIIB*, 4% *spnD39IIID* and 71% *spnD39IIIE*; Fig. 4h). In contrast, the pneumococci reisolated from mice showed a clear change in their SpnIIID39 allele type, with samples at 4 and 30 h after challenge having changed to a predominantly SpnD39IIIA state (Fig. 4h), clearly indicating selection for specific *spnD39III* alleles during infection^20^. Most striking was the change away from SpnIIID39E to SpnIIID39A in all of the blood samples from mice infected with the SpnD39IIIE-dominated inoculum. This suggests a definitive selection for the SpnD39IIIA allele in blood even as early as 4 h after challenge. Variations in allele frequency compared with inoculum were not detected in nasopharyngeal samples from the carriage experiment (Fig. 4g), indicating there is no such selection for alternate SpnD39III alleles in this environment. For these data, we confirmed that when allele quantification was performed directly on all nasal lavage samples, it yielded substantially the same allele composition as when testing first passage bacterial colonies grown from these samples (Supplementary Table 5).

## Discussion

Here, we have described a genetic switch that results in the presence of six different bacterial subpopulations each with distinct Type I RM target specificities and distinct epigenetic profiles. Such switchable Type I systems have previously been described, but these reports did not provide evidence for differential methylation or for phenotypic impact^6^,^11^,^12^. The SpnD39III system is distinct from the absolute ON/OFF switching of the Type III RM regulatory systems described in Gram-negative bacterial pathogens that switch between methylated and unmethylated states^21^; however, it does fit the definition of a phase variable regulon (‘phasevarion’)^4^,^22^. Importantly, the pneumococcal subpopulations identified in the present study exhibit phenotypic changes, including opacity phase variation differences, which have a major impact on bacterial virulence. This system provides a contingency mechanism for adaptation to changing environments^21^, such as those that are encountered during progression from asymptomatic colonization to invasive pneumococcal disease. Indeed, the SpnD39III system appears to be a central regulatory mechanism governing the fitness of the pneumococcus in distinct host niches. Since the *spnD39III* allele composition of a pneumococcal population significantly influences important phenotypes, and can also change rapidly, it is essential that all previous and future *in vitro* and *in vivo* studies should be interpreted in the context of this potential for switching between these heretofore undetected and uncharacterized differentiated pneumococcal subpopulations. We believe these findings represent a new paradigm in gene regulation in bacteria and therefore are of great significance to the infectious disease field.

## Methods

### Nomenclature of the RM systems identified

The well-described pneumococcal enzyme DpnI, originally clonded from a non-encapsulated D39 derivative^23^,^24^, will be named in this paper SpnD39ORF1631P (Table 1), to comply with current practice to use for each strain producing restriction enzymes an unique acronym. The other two Type II RM systems are annotated in the genome sequence of *S. pneumoniae* strain R6 (GenBank nucleotide database accession code AE007317) as SpnI (Restriction Enzyme database (REBASE) annotation SpnD39ORF1260P) and SpnII (REBASE annotation SpnD39ORF1079AP)^7^. This led us to annotate the phase variable Type I RM system as SpnD39III (SpnD39ORF454P) (GenBank accession codes KJ955483-6 and KJ398403-4). Wherever possible, we have followed the nomenclature outlined in the REBASE for *S. pneumoniae* D39 (ref. 9). Recombinant variants of the specificity subunit S.SpnD39III will be referred to using the suffix A–F. The second Type I RM system of D39 is not functional and since we have characterized it in strains WCH16 and WCH43, we have named the relevant variants S.SpnWCH16IVA (GenBank accession code KM030255) and SpnWCH43IVB (GenBank accession code KM030256). For the Type IV RM system, the only one currently without proven function, we have maintained the denomination SpnD39McrBCP already in REBASE^9^.

### Ethics statement

Animal experiments performed in Italy were approved by the Comitato Etico dell’Azienda Ospedaliera Universitaria Senese (Ethics Committee of the University Hospital of Siena, Siena, Italy). The animal experiments performed in Australia were approved by the University of Adelaide Animal Ethics Committee. The animal experiments performed in the UK were approved by the University of Leicester Ethics Committee in accordance with the U.K Home Office. All experiments were done in accordance with respective national and institutional guidelines.

### Bacterial strains and growth conditions

All pneumococcal strains were derived from strain D39 (serotype 2) and were routinely cultured on Tryptic Soy Broth agar plates with 3% v/v defibrinated horse blood at 37 °C in a 5% CO_2_ incubator. For colony morphology, the analysis plates contained 200 U ml^−1^ of catalase (Sigma, Germany) instead of blood. *E. coli* DH5α cells were grown according to standard protocols. The *Streptococcus–E. coli* shuttle vector pDP28 (GenBank accession code KJ395591) (ref. 25) was selected in *E. coli* using 10 μg ml^−1^ of chloramphenicol (Sigma) and in pneumococci using 5 μg ml^−1^ of erythromycin (Sigma).

### Construction of mutants

Recombinant D39 derivatives included a series of strains which stably expressed only one of the six *spnD39III* variants (*spnD39IIIA-F*) with a deletion of the truncated *hsdS* genes (S1.SpnD39III and S2.SpnD39III). Strains carrying either pDP28 (GenBank accession code KJ395591) or its derivatives pMRO1, pMRO2, or pMRO3 (see below) were derived from the SpnD39IIIA-D variant strains. All pneumococcal mutants were made following a multi-layer plating protocol for transformation as described elsewhere^26^. The SpnD39III variants were originated by transforming PCR generated fragments into naturally competent pneumococcal cells. In brief, flanking segments of the gene to be deleted were amplified with primers having 20 bp tails complementary to an *add9* spectinomycin resistance cassette. Reamplification of the flanking fragments together with the resistance cassette allowed synthetic constructs to be produced by PCR. Following transformation, representative transformants, selected using 200 μg ml^−1^ of spectinomycin (Sigma), were chosen. To construct pneumococcal mutants, the primers used included: 5′-GCAGTCTAAGCCATCAAATAC-3′ and 5′-GATCCACTAGTTCTAGAGCTTTCTGCCTGTAATTGTTCATC-3′ for the upstream flanking segment; 5′-GTATCGCTCTTGAAGGGAACACTTCGGCGATTTTCTGA-3′ and 5′-CGTGCGGTGGAATTTCTAT-3′ for the downstream flanking segment for the *spnD39IIIA* and *spnD39IIID* variants; 5′-GTATCGCTCTTGAAGGGAAGAGCATGTAGAAATCGGTTAT-3′ and 5′-TAATGCTTAAATCGCCCTTCT-3′ for the downstream flanking segment for the *spnD39IIIB* and *spnD39IIIC* variants; 5′-GGTGTTAGAATTATACGTGGTGG-3′ and 5′-GTATCGTCTTGAAGGGAACATTAAATAGTACCAGTATCTCCG-3′ for the downstream flanking segment for the *spnD39IIIE* and *spnD39IIIF* variants; 5′-GTATCGCTCTTGAAGGGAAGCCATCGTTTGGTCTACTAAGATGT-3′ and 5′-AGCATATCGCTTACGAAGAATACTT-3′ for the downstream flanking segment for the SpnD39III deletion mutant; and 5′-GCTCTAGAACTAGTGGATC-3′ and 5′-TTCCCTTCAAGAGCGATAC-3′ for the *aad9* cassette. Constructs were confirmed by Sanger sequencing and in the case of the SpnD39IIIA-D variant strains also by whole-genome Illumina sequencing (Institute of Applied Genomics, Udine, Italy). The sequences of the *spnD39III* loci in the locked mutant strains have been deposited in the GenBank nucleotide database with accession codes KJ955483 to KJ955486, KJ398403 and KJ398404). For construction of pDP28 derivatives the plasmid was originally extracted from *E. coli* strain DH5α (HiSpeed Plasmid Purification, Qiagen). Site-directed mutagenesis by inverted PCR was performed on pDP28 using primers 5′-CACCAAATGTAGCACCTGAAAGCAAATTCGACCCGGT-3′ and 5′-CAGGTGCTACATTTGGTGCCGCTTATTATCACTTATTCAGG-3′ to construct pMRO1, primers 5′-CACCACGGTCACACTGAAAGCAAATTCGACCCGGT-3′ and 5′-CAGTGTGACCGTGGTGCCGCTTATTATCACTTATTCAGG-3′ to construct pMRO2 and primers 5′-CCAGAACCTCTTACGTGGGTTCCAACTTTCACCATAATG-3′ and 5′-CACGTAAGAGGTTCTGGGCCGATCAACGTCTCATT-3′ to construct pMRO3. The modified plasmids were then transformed into *E. coli* by standard methods.

### Transformation of pneumococci

Transformation experiments to demonstrate methylation and restriction were performed using the same transformation protocol as above^26^. Plasmid pDP28 (extracted from *E. coli* DH5α as above) and its derivatives pMRO01, pMRO02 and pMRO03 (Supplementary Fig. 4) were transformed into pneumococcal variant strains SpnD39IIIA-D. Plasmids were then reextracted using an alkaline lysis protocol, as described elsewhere^27^, and each retransformed into the locked strains. The quantity of plasmid used for transformation was 10 ng of plasmid DNA for 100 μl of competent cells.

To test chromosomal insertion of linear PCR fragments during transformation (Supplementary Fig. 2f), the mutant gene SPD_0661 containing a kanamycin cassette (*aphIII*) (ref. 28) was amplified using the primers 5′-CGGTAAGGCTTTGATGGTAGTTA-3′ and 5′-GGTTTACCTTCAAGACTTACTGTG-3′. This fragment contained one SpnD39IIIA recognition site. Modified fragments were constructed with two SpnD39IIIA sites in all the possible orientations: co-directional, non-co-directional in tail-to-tail orientation and non-co-directional in head-to-head orientation. This mutagenesis was performed using the primers 5′-CGAATGTAGCACCTGAGCTGGGGATCCGTTTGAT-3′ and 5′-CAGGTGCTACATTCGTGAACCTGAGATAATCCCTACG-3′ for co-directional site orientation, 5′-CAGGTGCTACATTCGAGCTGGGGATCCGTTTGAT-3′ and 5′-CGAATGTAGCACCTGTGAACCTGAGATAATCCCTACG-3′ for tail-to-tail sites and 5′-CAGGTGCTACATTCGGCCTACGAGGAATTTGTATCTTC-3′ and 5′-CGAATGTAGCACCTGGCTCGGGACCCCTATCTAGCGA-3′ for head-to-head oriented sites. The quantity of PCR DNA used for transformation was 100 ng of DNA for 100 μl of competent cells and transformants were selected with 500 μg ml^−1^ of kanamycin.

### Allele quantification

The variant alleles of *hsdS* in wild-type D39 were quantified utilizing an allele scan protocol (Supplementary Fig. 6). The whole *hsdS* locus was PCR amplified (4.2 kb) from extracted genomic DNA utilizing primers 5′-CCATTATCTATAGGCGTATTTTTACG3′- and FAM–5′-GGAAACTGAGATATTTCGTGGTG-3′ (where FAM is 6-fluorescein amidite). The PCR products were then digested with both DraI and PleI (New England Biolabs, MA, USA). This digestion was predicted to yield different sized FAM-labelled fragments for each of the variant forms. The pool of restriction fragments was run on an ABI prism Gene Analyser (Life Technologies). The area of the peak given by each labelled fragment, each corresponding to the prevalence of one of the variant forms, was quantified using Peak Scanner v1.0.

### Genome and gene expression analysis

The genomic analysis that allowed the identification of the SpnD39III system was performed using Artemis Comparison Tool utilizing *S. pneumoniae* D39 (GenBank nucleotide database accession code NC_008533.1) and TIGR4 (accession code NC_003028.3) genome sequences. Whole-genome sequencing was performed for strains SpnD39IIIA-D (Institute of Applied Genomics, Udine, Italy) using an Illumina Genome Analyzer II platform (Illumina, San Diego, CA). Analysis of these sequences was performed using the programs FastQC for analysis of the quality of the reads, Trimmomatic (version 0.30) DynamicTrim for quality improvements, Mosaik and SamTools for alignment and VarScan for detection of SNPs, insertions and deletions. For gene expression, pneumococcal strains were grown to mid-log phase (OD_590_ approximately 0.15). 2 ml of cells were harvested by centrifugation at 10,000 r.p.m. for 15 min and resuspended in 90 μl of TE with 10 μl of lysozyme. The NucleoSpinRNA II kit (Macherey-Nagel, Germany) was used for RNA extraction following the manufacturer’s protocol. Frozen RNA samples were sent to the Institute of Applied Genomics (University of Udine, Italy) for RNA-seq analysis using an Illumina Genome Analyzer II platform (Illumina). RNA-Seq fastq files were trimmed using ERNE, read mapping to the reference genome D39 was performed using Tophat, and transcript abundance and differential expression analyses were carried out using Cufflinks and the R package cummeRbund. Three independent replicas were used for each sample. The RNA-seq data were deposited in the NCBI GEO database with accession code GSE55182.

### Methylome analysis

DNA was extracted from overnight cultures in TSB from each of the different variants using the High Pure PCR Template Preparation kit (Roche, Italy) and sent to Pacific Biosciences (Menlo Park, CA, USA) where methylome data was obtained by SMRT. SMRTbell libraries were prepared as previously described^29^. Briefly, gDNA was sheared to an average length of approximately 10 kb using g-TUBEs (Covaris, Woburn, MA, USA), treated with DNA damage repair mix, end repaired and ligated to hairpin adapters. Incompletely formed SMRTbell templates were digested using Exonuclease III (New England Biolabs) and Exonuclease VII (Affymetrix, OH, USA). Sequencing was carried out on the *PacBio RS II* (Menlo Park, CA, USA) using standard protocols for long insert libraries. Methylation sites of variants SpnIIIA-C were experimentally confirmed by protection of pDP28 DNA from digestion by methylation sensitive enzymes with overlapping target specificity. Plasmid DNA for these experiments was extracted from strains expressing a single SpnD39III variant and shown by SMRT sequencing to methylate one single SpnD39III target. When extracted from pneumococcal strains, a manual protocol of alkaline lysis was used. Plasmid pDP28 was originally extracted from an *E. coli* DH5α strain using the HiSpeed Plasmid Midi Kit from Qiagen (Italy). Enzymes used included AcuI (5′-CTGAAG-3′) whose target sites can overlapp SpnIIIA target sites; SfuI (5′-TTCGAA-3′), overlapping SpnIIIB; and BsaA1 (5′-TACGTG-3′), overlapping SpnIIIC (all from Fermentas, Germany). Protection from cleavage was visualized on ethidium bromide stained agarose gels.

### Uronic acid quantification

Pneumococcal capsule samples were prepared as previously described^30^. In brief, deoxycolate-lysed pneumococci were treated by adding 100 U of mutanolysin (Sigma, Australia), 50 U DNaseI (Roche, Australia) and 50 μg RNaseA and incubating overnight at 37 °C, followed by treatment with 100 μg of proteinase K at 56 °C for 4 h. Uronic acid was then quantitated colourimetrically, as described previously^30^.

### Quantitative western blot

Relative expression of LuxS was determined by quantitative western blot analysis of whole-cell lysates. The total protein in the lysates was determined using the BCA Protein Assay kit (Thermo Scientific, Australia). Bands on western blots were detected uisng anti-LuxS polyclonal murine antiserum at a dilution of 1:2,000 and donkey anti-mouse IRDye 800 CW secondary antibody (LI-COR Biosciences, USA) at a dilution of 1:50,000. The blot was scanned and LuxS expression was quantified using the Odyssey Infrared Imaging System (LI-COR Biosciences).

### Macrophage phagocytosis

Standard phagocytosis assays were performed as previously described^31^. Spleen and bone marrow macrophages were isolated from mice using a modified protocol^20^. RAW 264.7 were cultured in RPMI medium (Defined Hyclone, Logan, UT, USA) supplemented with 10% heat inactivated fetal calf serum. At confluence of 90%, 0.1 ml of pneumococcus cultured to OD_590_ 0.25 were added. After 45 min plates were washed and reincubated with 10 mg l^−1^ of penicillin and 200 mg ml^−1^ of gentamicin (Sigma, Germany) for 30 min. Intracellular bacteria were enumerated after lysis with saponin 1%.

### Experimental infections

Carriage experiments for variants SpnD39IIIA-D were performed in Siena, Italy, with mice from Charles River (Italy). To evaluate nasopharyngeal carriage, 15 groups of five female, 7 weeks old, BALB/c mice (resistant to systemic pneumococcal infection) were infected intranasally (5 × 10^4^ c.f.u. in 10 μl PBS)^32^. Pneumococcal strains included the D39 wild type and the strains expressing a single SpnD39III variant (SpnD39IIIA, SpnD39IIIB, SpnD39IIIC or SpnD39IIID). At the time of killing (days 1, 3 and 7), nasal lavages were performed. An equivalent protocol was followed for variants SpnD39IIIE and F (using wild-type D39 as control), when performing intranasal infection at the University of Leicester, UK, using groups of five female, 9 weeks old, BALB/c mice supplied by Harlan (Bicester, UK). For the invasive disease model, performed in Adelaide, Australia, female outbred 6-week-old CD-1 mice were inoculated intravenously with 1 × 10^5^ c.f.u. of the same pneumococcal strains as above (100 μl inoculum)^33^,^34^. Groups of 12 mice were inoculated for each strain and blood was collected by cheek bleeding at 4 and 30 h post infection. At 30 h, spleen, brain and liver samples were also taken. Statistical significance was calculated on log-transformed data using the unpaired (two-tailed) *t*-test.

### Mathematical analysis of target site distribution

Comparison of number of target sites (markers): by using nucleotide frequencies, we can estimate the expected number of each marker using the assumption that nucleotides are distributed independently (that is, the probability of finding a specific nucleotide in a specified position does not depend on nucleotides in other positions). We tested the significance of the differences between expected and observed numbers for each marker site in both strands and in each strand separately by *χ*^2^-test. To analyse the positional relationship between markers, we compared the empirical distribution of distances with the uniform distribution for relatively small distances (less than half of the average distance). For this comparison, we calculated the cumulative distribution function for distances at the selected scale and used the Kolmogorov–Smirnov test^35^. For the distances between the markers situated in different strands, we used several samplings: the distances from the marker in the leading strand to the downstream marker in the lagging strand (‘tail-to-tail’), the distances from the marker in the leading strand to the upstream marker in the lagging strand (‘head-to-head’) and the distances between markers in different strands, for both ‘tail-to-tail’ and ‘head-to-head’ orientations (‘bidirectional’). If the empirical cumulative density significantly exceeded the corresponding uniform distribution at the selected scale, then we concluded that the markers demonstrated attraction over the short distance (prevalence of short distances). For additional validation of this test, we use direct simulation. We also applied the Clark–Evans test^36^ and compare the average distance to the nearest neighbours with the average for Poisson distribution (that is 1/(2*r*), where *r* is the density of markers) with the proper modification for oriented pairs of markers in different strands.

## Additional information

**Accession codes**: Gene expression (RNA-seq) data were deposited in the NCBI GEO database with accession code GSE55182. The sequences of the *spnD39III* loci in the locked mutant strains were deposited in the GenBank nucleotide database with accession codes KJ955483, KJ955484, KJ955485, KJ955486, KJ398403 and KJ398404.

**How to cite this article**: Manso, A. S. *et al.* A random six-phase switch regulates pneumococcal virulence via global epigenetic changes. *Nat. Commun.* 5:5055 doi: 10.1038/ncomms6055 (2014).

## Supplementary Material

Supplementary Information Supplementary Figures 1-6, Supplementary Tables 1-5 and Supplementary Reference

## Figures and Tables

**Table 1:** RM systems of pneumococcal strain D39.

**RM system name**	**Type**	**ORFs** *	**Specificity** †	**TRD**	**Modified base**	**Number of sites** *	**Predicted no. of sites** *	**Difference (%)**	**Strand specificity (%)** ‡
SpnD39ORF1631P (DpnI)	Type II	SPD_1630-1	5′-GATC-3′3′-CTAG-5′	—	—	7,164	7,324	−2	—
SpnD39I (SpnD39ORF1260P)	Type II	SPD_1259-60	5′-TCTAG**A**-3′ 3′-**A**GATCT-5′	—	^m6^A	644	438	47§	—
SpnD39II|| (SpnD39ORF1079AP)	Type II	SPD_1079-80	5′-TCG**A**G-3′ 3′-AGCTG-5′	—	^m6^A	1,509	1454	4	—
SpnD39IIIA (SpnD39ORF454P)	Type I	SPD_0450-5	5′-CRA**A**N_8_CTG-3′3′-GYTTN_8_G**A**C-5′	1.1, 2.1	^m6^A	720	438	64§	66§
SpnD39IIIB	Type I	SPD_0450-5	5′-CRA**A**N_9_TTC-3′3′-GYTTN_9_**A**AG-5′	1.1, 2.2	^m6^A	1,029	665	55§	64§
SpnD39IIIC	Type I	SPD_0450-5	5′-C**A**CN_8_TTC-3′3′-GTGN_8_**A**AG-5′	1.2, 2.2	^m6^A	641	876	−27§	66§
SpnD39IIID	Type I	SPD_0450-5	5′-C**A**CN_7_CTG-3′3′-GTGN_7_G**A**C-5′	1.2, 2.1	^m6^A	428	577	−26§	67§
SpnD39IIIE	Type I	SPD_0450-5	5′-CRA**A**N_8_CTT-3′3′-GYTTN_8_G**A**A-5′	1.1, 2.3	^m6^A	1,028	665	55§	63§
SpnD39IIIFP	Type I	SPD_0450-5	5′-C**A**CN_7_CTT-3′3′-GTGN_7_G**A**A-5′	1.2, 2.3	^m6^A	796	876	−9§	64§
SpnD39ORF782P	Type I	SPD_0782-4¶	not active; see below	—	—	—	—	—	—
SpnD39McrBCP	Type IV	SPD_1108-9	not known	—	—	—	—	—	

ORF, open reading frame; RM, restriction-modification system; TRD, target recognition domain.

Using strains WCH16 and WCH43 (ref. 14), we identified the target sites of the two possible variants of this Type I RM system to be 5′-G(^m6^A)YN_6_TATC-3′ and 3′-CTRN_6_(^m6^A)TAG-5′ for S.SpnWCH16IVA (GenBank nucleotide database accession code KM030255) and 5′-TG(^m6^A)N_7_TATC-3′ and 3′-ACTN_7_(^m6^A)TAG-5′ for S. SpnWCH43IVB (GenBank code KM030256).

^*^Open reading frame (ORF) numbering and calculations are done for strain D39 (GenBank nucleotide database accession code CP000410.1).

^†^Methylated adenine in bold underlined.

^‡^Percentage of sites mapping to the lagging strand of the genome (3′-5′ strand from start to 1,047,526 and 5′-3′ from 1,047,527 to end).

^§^Differences were analysed by *χ*^2^ analysis and found to be significant for all values (*P*<0.001).

^||^The target site TCG(^m6^A)G could be associated to Spn39II due to absence of both this locus and TCG(^m6^A)G methylation in strain WCH43 (ref. 14).

^¶^The gene encoding S.SpnD39ORF782P is truncated in D39 and no methylation was detected.

**Table 2:** Differential expression in the SpnD39IIIB variant strain relative to the other SpnD39III variants*.

**D39 ORF**	**Gene**	**Description**	**SpnIIIA**	**SpnIIIC**	**SpnIIID**
SPD_0309	* luxS *	LuxS S-ribosylhomocysteine lyase	−4.9	−4.2	−3.8
SPD_0310	(unnamed)	Uncharacterized protein	−4.5	−4.1	−4.0
SPD_0311	* dexB *	Glucan 1,6-alpha-glucosidase	−3.4	−4.6	−4.5
SPD_0315	* cps2A *	LCP capsule anchoring protein	−4.0	−4.3	−4.2
SPD_0316	* cps2B *	Tyrosine-protein phosphatase	−3.1	−3.2	−3.1
SPD_0317	* cps2C *	Polysaccharide transport protein	−2.5	−2.7	−2.6
SPD_0318	* cps2D *	Tyrosine-protein kinase	−2.1	−2.6	−2.5
SPD_0321	* cps2F *	Glycosyl transferase	−2.3	−2.7	−2.6
SPD_0322	* cps2G *	Glycosyl transferase	−2.4	−2.8	−3.3
SPD_0323	* csp2H *	Polysaccharide polymerase	−2.2	−2.6	−2.9
SPD_0325	* cps2J *	Membrane protein	−2.9	−3.0	−3.9
SPD_0326	* cps2K *	UDP-glucose 6-dehydrogenase	−2.9	−2.7	−3.0
SPD_0327	* cps2P *	UDP-galactopyranose mutase	−2.6	−3.3	−3.9
SPD_0328	* cps2L *	Glucose-1-P thymidylyltransferase	−2.3	−3.2	−4.0
SPD_0330	* rfbB *	dTDP-glucose 4,6-dehydratase	−2.1	−3.3	−4.1
SPD_0331	* rfbD *	dTDP-4-dehydrorhamnose reductase	−2.1	−3.0	−3.7
SPD_0332	(unnamed)	Uncharacterized protein	−2.5	−3.4	−3.5

^*^Log2 fold change in gene expression as assessed by RNA-seq. Data are deposited in the NCBI GEO database with accession code GSE55182.

**Table 3:** Differential expression in the SpnD39IIIA variant strain*.

**D39 ORF**	**Gene**	**Description**	**SpnIIIB**	**SpnIIIC**	**SpnIIID**
SPD_0257		Uncharacterized protein	2.4	2.2	2.6
SPD_0286		Glutathione peroxidase	2.4	2.7	2.2
SPD_0460	* dnaK * †	Chaperone protein DnaK	2.8	2.5	2.7
SPD_0473	* blpY *	Immunity protein BlpY	−5.0	−4.6	−3.3
SPD_0474		Uncharacterized protein	−5.6	−4.8	−3.1
SPD_0475		CAAX protease	−5.3	−4.5	−2.9
SPD_0675		Uncharacterized protein	−3.6	−2.9	−2.1
SPD_0708		Haloacid dehalogenase-like hydrolase	−2.0	−2.6	−2.5
SPD_1256		Peptidase, U32 family protein	−3.4	−3.0	−3.9
SPD_1307	†	Uncharacterized protein	−2.0	−2.2	−2.2
SPD_1415		Pyrimidine nucleotide sulfite reductase	2.3	2.7	3.6
SPD_1452		Uncharacterized protein	−2.4	−2.3	−2.4
SPD_1461	* psaB *	Manganese ABC transporter, ATP binding	2.8	2.9	2.6
SPD_1462	* psaC *	Manganese ABC transporter, permease	3.0	3.0	2.7
SPD_1463	* psaA *	Manganese, substrate-binding lipoprotein	2.6	2.9	2.7
SPD_1535	* scrR *	Sucrose operon repressor	−2.2	−2.1	−2.4
SPD_1992		PTS system, IIA component	−2.7	−2.6	−2.4
SPD_1993	* fucU *	L-fuculose transport protein	−2.2	−2.6	−2.2
SPD_1994	* fucA *	L-fuculose phosphate aldolase	−2.2	−2.5	−2.0
SPD_1995	* fucK *	L-fuculose kinase FucK	−2.4	−2.6	−2.4
sRNA_F26		RNA downstream *livJ*	2.4	2.2	2.3
sRNA_SN46		RNA upstream *pcpA*	2.4	3.5	2.5

Data are deposited in the NCBI GEO database with accession code GSE55182.

^*^Log2 fold change in gene expression as assessed by RNA-seq.

^†^A SpnD39IIIA site maps in a non-coding RNA preceding the gene.

## References

[b1] Donati C. *et al.* Structure and dynamics of the pan-genome of *Streptococcus pneumoniae* and closely related species. Genome Biol. 11, R107 (2010). 21034474 10.1186/gb-2010-11-10-r107 PMC3218663

[b2] Webster L. T. & Clow A. D. Intranasal virulence of pneumococci for mice. J. Exp. Med. 58, 465–483 (1933). 19870209 10.1084/jem.58.4.465 PMC2132311

[b3] Weiser J. N., Austrian R., Sreenivasan P. K. & Masure H. R. Phase variation in pneumococcal opacity: relationship between colonial morphology and nasopharyngeal colonization. Infect. Immun. 62, 2582–2589 (1994). 8188381 10.1128/iai.62.6.2582-2589.1994 PMC186548

[b4] Srikhanta Y. N., Fox K. L. & Jennings M. P. The phasevarion: phase variation of type III DNA methyltransferases controls coordinated switching in multiple genes. Nat. Rev. Microbiol. 8, 196–206 (2010). 20140025 10.1038/nrmicro2283

[b5] Loenen W. A., Dryden D. T., Raleigh E. A. & Wilson G. G. Type I restriction enzymes and their relatives. Nucleic Acids Res. 42, 20–44 (2014). 24068554 10.1093/nar/gkt847 PMC3874165

[b6] Tettelin H. *et al.* Complete genome sequence of a virulent isolate of *Streptococcus pneumoniae*. Science 293, 498–506 (2001). 11463916 10.1126/science.1061217

[b7] Hoskins J. *et al.* Genome of the bacterium *Streptococcus pneumoniae* strain R6. J. Bacteriol. 183, 5709–5717 (2001). 11544234 10.1128/JB.183.19.5709-5717.2001 PMC95463

[b8] Lacks S. A., Mannarelli B. M., Springhorn S. S. & Greenberg B. Genetic basis of the complementary DpnI and DpnII restriction systems of *S. pneumoniae*: An intercellular cassette mechanism. Cell 46, 993–1000 (1986). 3019562 10.1016/0092-8674(86)90698-7

[b9] Roberts R. J., Vincze T., Posfai J. & Macelis D. REBASE-a database for DNA restriction and modification: enzymes, genes and genomes. Nucleic Acids Res. 38, D234–D236 (2010). 19846593 10.1093/nar/gkp874 PMC2808884

[b10] Fuller-Pace F. V., Bullas L. R., Delius H. & Murray N. E. Genetic recombination can generate altered restriction specificity. Proc. Natl Acad. Sci. USA 81, 6095–6099 (1984). 6091134 10.1073/pnas.81.19.6095 PMC391866

[b11] Cerdeno-Tarraga A. M. *et al.* Extensive DNA inversions in the *B. fragilis* genome control variable gene expression. Science 307, 1463–1465 (2005). 15746427 10.1126/science.1107008

[b12] Dybvig K., Sitaraman R. & French C. T. A family of phase-variable restriction enzymes with differing specificities generated by high-frequency gene rearrangements. Proc. Natl Acad. Sci. USA 95, 13923–13928 (1998). 9811902 10.1073/pnas.95.23.13923 PMC24968

[b13] Fang G. *et al.* Genome-wide mapping of methylated adenine residues in pathogenic *Escherichia coli* using single-molecule real-time sequencing. Nat. Biotechnol. 30, 1232–1239 (2012). 23138224 10.1038/nbt.2432 PMC3879109

[b14] Mahdi L. K., Ogunniyi A. D., LeMessurier K. S. & Paton J. C. Pneumococcal virulence gene expression and host cytokine profiles during pathogenesis of invasive disease. Infect. Immun. 76, 646–657 (2008). 18039836 10.1128/IAI.01161-07 PMC2223468

[b15] Meisel A., Bickle T. A., Kruger D. H. & Schroeder C. Type III restriction enzymes need two inversely oriented recognition sites for DNA cleavage. Nature 355, 467–469 (1992). 1734285 10.1038/355467a0

[b16] Lobry J. R. & Louarn J. M. Polarisation of prokaryotic chromosomes. Curr. Opin. Microbiol. 6, 101–108 (2003). 12732297 10.1016/s1369-5274(03)00024-9

[b17] Johnston C., Martin B., Granadel C., Polard P. & Claverys J. P. Programmed protection of foreign DNA from restriction allows pathogenicity island exchange during pneumococcal transformation. PLoS Pathog. 9, e1003178 (2013). 23459610 10.1371/journal.ppat.1003178 PMC3573125

[b18] Avery O. T. & Dubos R. The protective action of a specific enzyme against type III pneumococcus infection in mice. J. Exp. Med. 54, 73–89 (1931). 19869903 10.1084/jem.54.1.73 PMC2132045

[b19] King S. J. *et al.* Phase variable desialylation of host proteins that bind to *Streptococcus pneumoniae in vivo* and protect the airway. Mol. Microbiol. 54, 159–171 (2004). 15458413 10.1111/j.1365-2958.2004.04252.x

[b20] Gerlini A. *et al.* The role of host and microbial factors in the pathogenesis of pneumococcal bacteraemia arising from a single bacterial cell bottleneck. PLoS Pathog. 10, e1004026 (2014). 24651834 10.1371/journal.ppat.1004026 PMC3961388

[b21] Moxon R., Bayliss C. & Hood D. Bacterial contingency loci: the role of simple sequence DNA repeats in bacterial adaptation. Annu. Rev. Genet. 40, 307–333 (2006). 17094739 10.1146/annurev.genet.40.110405.090442

[b22] Srikhanta Y. N., Maguire T. L., Stacey K. J., Grimmond S. M. & Jennings M. P. The phasevarion: a genetic system controlling coordinated, random switching of expression of multiple genes. Proc. Natl Acad. Sci. USA 102, 5547–5551 (2005). 15802471 10.1073/pnas.0501169102 PMC556257

[b23] Lacks S. A., Mannarelli B. M., Springhorn S. S. & Greenberg B. Genetic basis of the complementary DpnI and DpnII restriction systems of *Streptococcus pneumoniae* - an intercellular cassette mechanism. Cell 46, 993–1000 (1986). 3019562 10.1016/0092-8674(86)90698-7

[b24] de la Campa A. G., Springhorn S. S., Kale P. & Lacks S. A. Proteins encoded by the DpnI restriction gene cassette. Hyperproduction and characterization of the DpnI endonuclease. J. Biol. Chem. 263, 14696–14702 (1988). 2844782

[b25] Oggioni M. R., Iannelli F. & Pozzi G. Characterization of cryptic plasmids pDP1 and pSMB1 of *Streptococcus pneumoniae*. Plasmid 41, 70–72 (1999). 9887308 10.1006/plas.1998.1364

[b26] Iannelli F. & Pozzi G. Method for introducing specific and unmarked mutations into the chromosome of *Streptococcus pneumoniae*. Mol. Biotechnol. 26, 81–86 (2004). 14734825 10.1385/MB:26:1:81

[b27] Birnboim H. C. A rapid alkaline extraction method for the isolation of plasmid DNA. Methods Enzymol. 100, 243–255 (1983). 6353143 10.1016/0076-6879(83)00059-2

[b28] Bidossi A. *et al.* A functional genomics approach to establish the complement of carbohydrate transporters in *Streptococcus pneumoniae*. PLoS ONE 7, e33320 (2012). 22428019 10.1371/journal.pone.0033320 PMC3302838

[b29] Clark T. A. *et al.* Characterization of DNA methyltransferase specificities using single-molecule, real-time DNA sequencing. Nucleic Acids Res. 40, e29 (2012). 22156058 10.1093/nar/gkr1146 PMC3287169

[b30] Morona J. K., Morona R. & Paton J. C. Attachment of capsular polysaccharide to the cell wall of *Streptococcus pneumoniae* type 2 is required for invasive disease. Proc. Natl Acad. Sci. USA 103, 8505–8510 (2006). 16707578 10.1073/pnas.0602148103 PMC1482522

[b31] Alatery A. & Basta S. An efficient culture method for generating large quantities of mature mouse splenic macrophages. J. Immunol. Methods 338, 47–57 (2008). 18675819 10.1016/j.jim.2008.07.009

[b32] Trappetti C. *et al.* Sialic acid: a preventable signal for pneumococcal biofilm, colonisation and invasion of the host. J. Infect. Dis. 199, 1497–1505 (2009). 19392624 10.1086/598483

[b33] Oggioni M. R. *et al.* Antibacterial activity of a competence-stimulating peptide in experimental sepsis caused by *Streptococcus pneumoniae*. Antimicrob. Agents Chemother. 48, 4725–4732 (2004). 15561850 10.1128/AAC.48.12.4725-4732.2004 PMC529211

[b34] Oggioni M. R. *et al.* Switch from planktonic to sessile life: a major event in pneumococcal pathogenesis. Mol. Microbiol. 61, 1196–1210 (2006). 16925554 10.1111/j.1365-2958.2006.05310.x PMC1618759

[b35] Wilcox R. Kolmogorov-Smirnov test. Encyclopedia of Biostat. 4, doi:10.1002/0470011815.b2a15064 (2005).

[b36] Clark P. J. & Evans F. C. Distance to nearest neighbor as a measure of spatial relationships in populations. Ecology 35, 445–453 (1954).

