# Supplementary material for: A random six-phase switch regulates pneumococcal virulence via global epigenetic changes
Source: Nat Commun. 2014 Sep 30;5:5055. doi: 10.1038/ncomms6055 (PMC4190663; doi:10.1038/ncomms6055)
Supplement: Supplementary Information — Supplementary Figures 1-6, Supplementary Tables 1-5 and Supplementary Reference [file ncomms6055-s1.pdf]

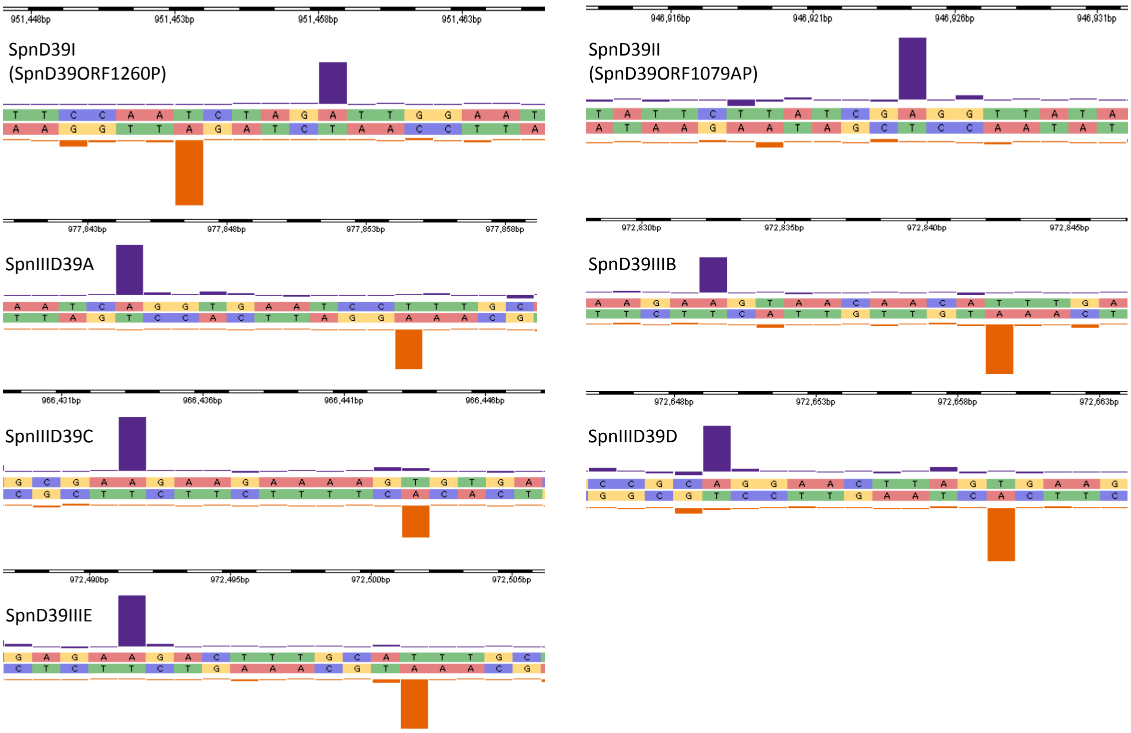

Supplementary Figure 1: DNA methylome analysis. Examples of methylation pattern detection for different target specificities in phenotype switching *S. pneumoniae* D30 and locked derivatives. The single sites shown are selected randomly on the genome. Each panel shows an instance of methylation imparted by the different methyltransferases, with the purple track representing the Interpulse Duration Ratio (IPD) of the forward DNA strand, and the orange track the IPD of the reverse DNA strand[1].

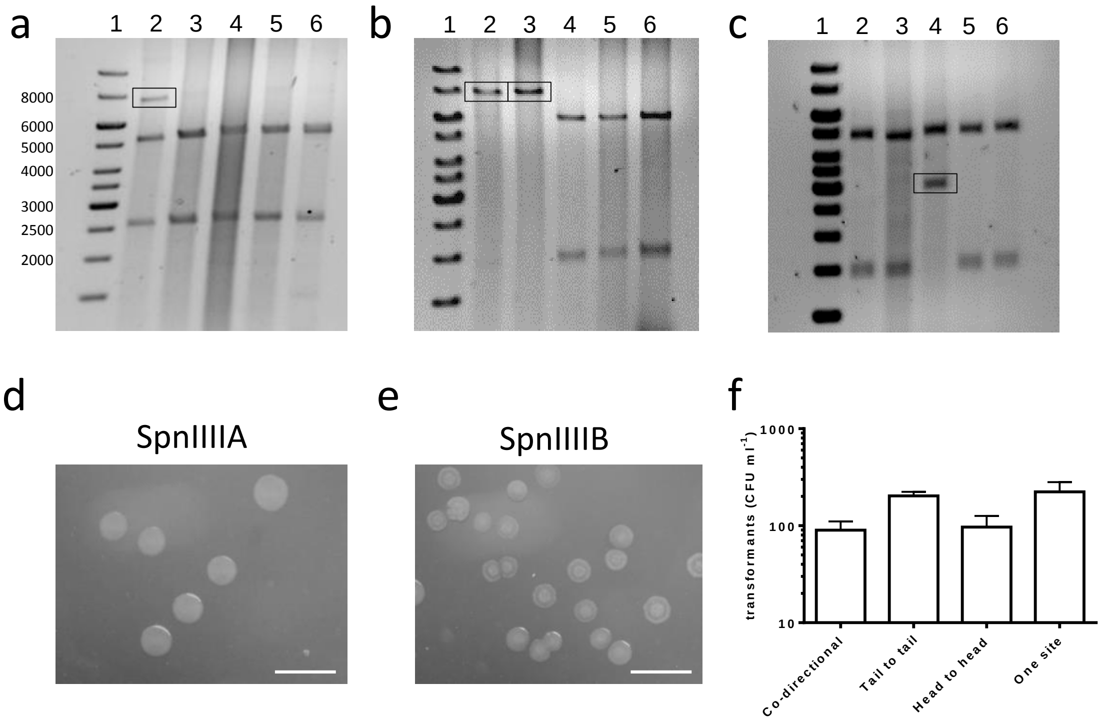

Supplementary Figure 2: Phenotypes conferred by SpnD39III variants. Methylation by SpnD39III was demonstrated by showing protection from cleavage with methylation sensitive enzymes. Plasmid pDP28 (Supplementary Fig. 4) was extracted from SpnD39III locked strains and cleaved with the methylation sensitive restriction enzymes AcuI (a), SfuI (b), or BsaA1 (c). Molecular weight standard (GeneRulerTM 1kb DNA Ladder) (lane 1), plasmid DNA extracted from strain expressing SpnD39IIIA (lane 2), SpnD39IIIB (lane 3), SpnD39IIIC (lane 4), SpnD39IIID (lane 5), and from a strain with a deletion of the SpnD39III locus (lane 6). Methylation of SpnD39IIIA (CAAATGTAGCACCTG; pos. 6011) protects partially from AcuI cleavage (CTGAAG) (panel a lane 2). Methylation by SpnD39IIIA (CGAAAAACCGCCCTG; pos. 6011) and SpnD39IIIB (CGAATTTCTGCCATTC; pos. 4033) protects from cleavage with SfuI (TTCGAA) (panel b, lane 2 and 3). Methylation by SpnD39IIIC (GAACCTCTTACGTG; pos. 4860) protects from cleavage with BsaA1 (TACGTG) (panel c, lane 4). The plasmid DNA fragments protected from restriction by methylation are boxed. Colony morphology of SpnD39IIIA colonies (panel d) and SpnD39IIIB variant colonies (panel e) are shown after 24 h growth on catalase agar plates. Scale bar, 1 mm. Efficiency of chromosomal transformation in D39 of a linear DNA fragment engineered to contain differently oriented SpnD39IIIA sites is shown in panel f. The mean of three different experimental replicas and the standard deviation are shown. Differences in transformation frequency in this panel analysed by a T-test are non-significant.

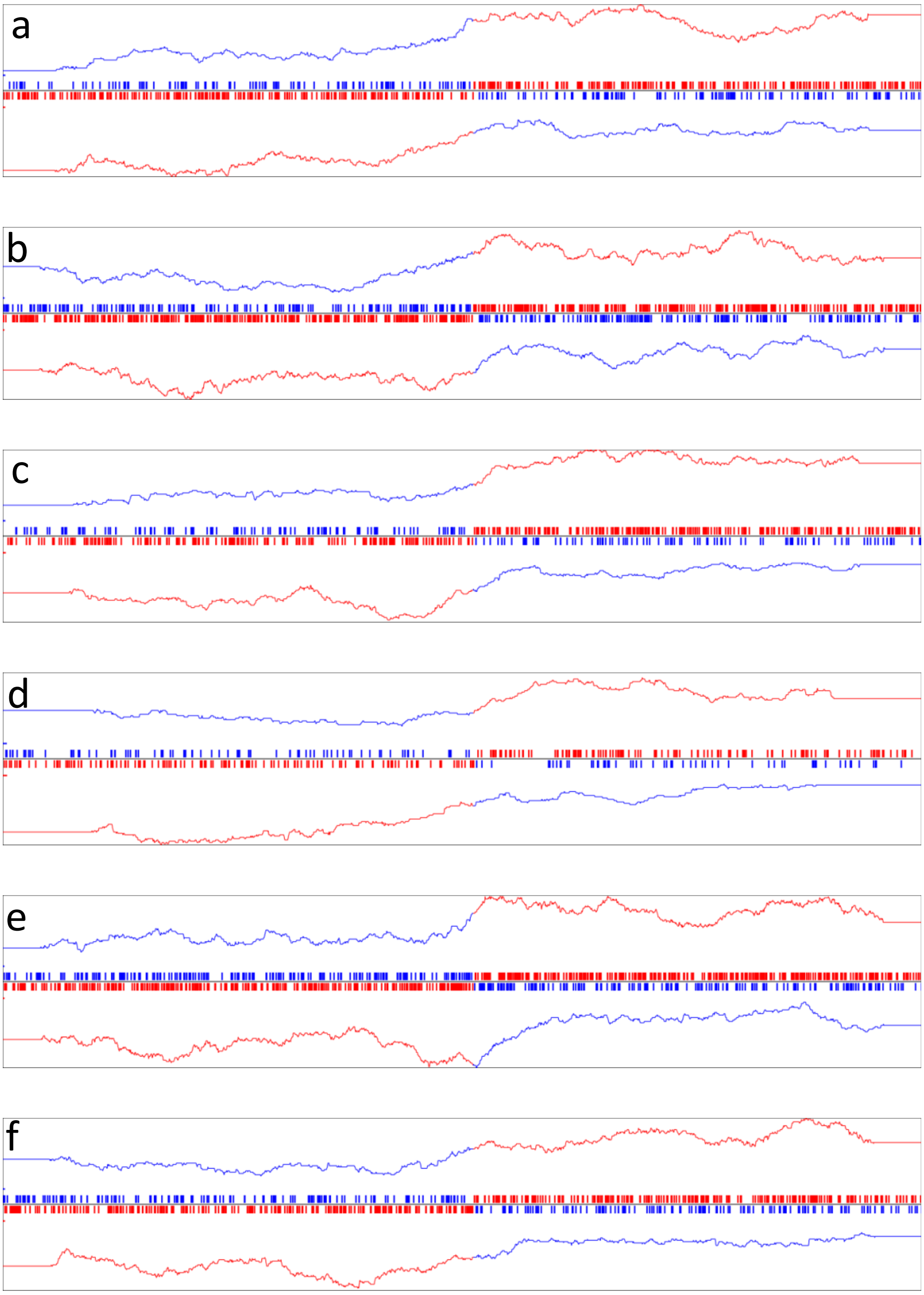

Supplementary Figure 3: Distribution of SpnD39IIIA-F sites (panels a-f respectively) in the genome of *S. pneumoniae* D39. Sites on the leading strand are in blue, and sites on the lagging strand in red. The relative density of sites is plotted as line above and below the sequences.

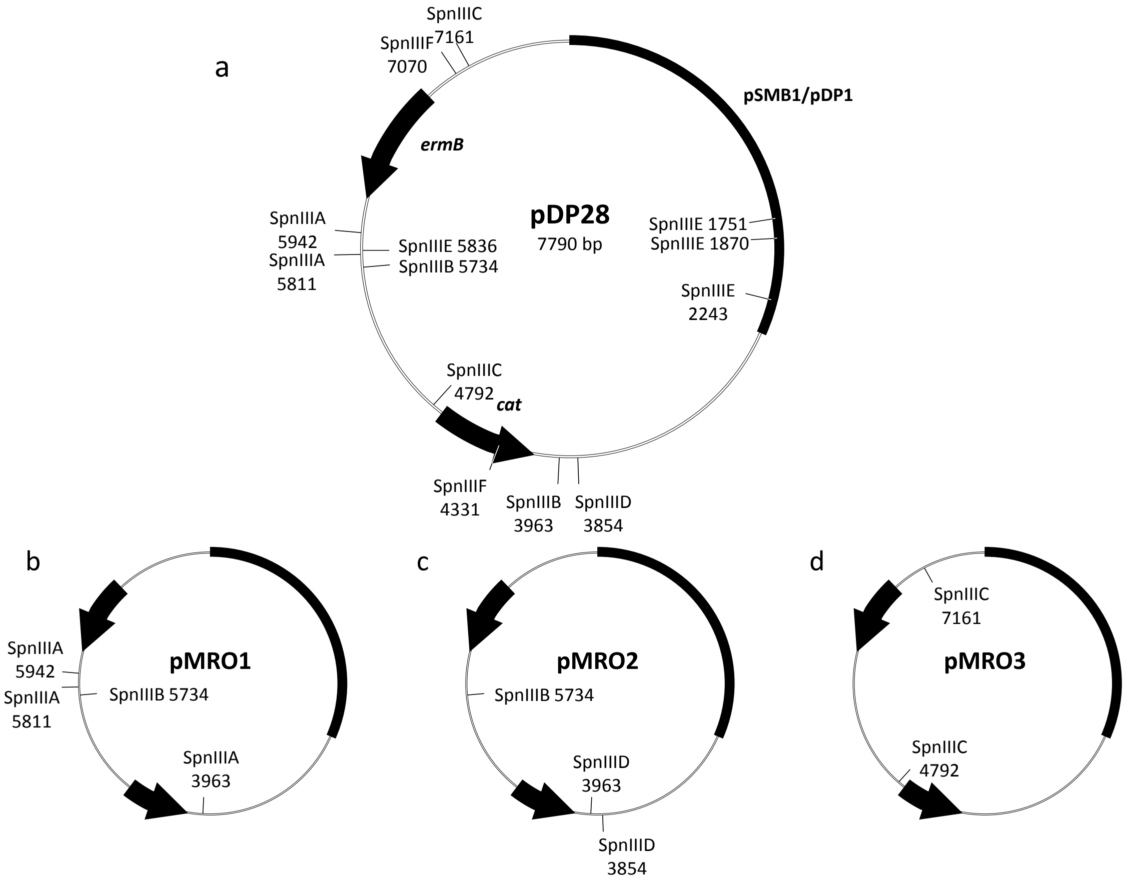

Supplementary Figure 4: Schematic map of the *S. pneumoniae* – *E. coli* shuttle vector pDP28 and its derivatives. Plasmid pDP28 (GenBank accession code KJ395591) is shown with the target sites of the six variant forms of SpnD39IIIA-F. Recombinant pDP28 derivatives are shown in panels b-d. In pMRO1 a SpnD39IIIA site was substituted for the SpnD39IIIB site in position 3963 (b), in pMRO2 a SpnD39IIID site was substituted for the same SpnD39IIIB site at 3963 (c) and in pMRO3 the SpnD39IIIC site in position 7161 was inverted (d). Positioning of the sites outside the circle indicates localisation on the leading strand and vice versa. The homology of pDP28 to the pneumococcal plasmid pDP1/pSMB1 (GenBank accession code AF047385)[15] is shown as a thick line. All relevant SpnD39III sites tested in the transformation experiments fall in the region of pDP28 that does not show homology to the resident plasmid pDP1 and therefore does not form a hemi-methylated dsDNA strand prior to replication.

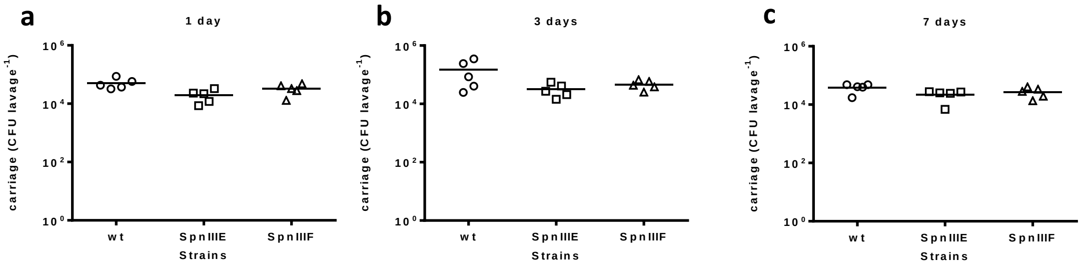

Supplementary Figure 5: *In vivo* phenotypes of SpnD39IIIE and SpnD39IIIF variant strains in carriage. D39 wt and derivatives expressing only the SpnD39IIIE or SpnD39IIIF variant were used in carriage experiments. BALB/c mice were intra-nasally infected with $5x10^4$ pneumococci. The extent of carriage was evaluated by nasal lavage at day 1 (panel a), day 3 (panel b) and day 7 (panel c). The data are presented separately from those in Figure 4 because change of animal supplier, housing facility and laboratory resulted in higher numbers of colonising pneumococci with respect to the data reported in Figure 4.

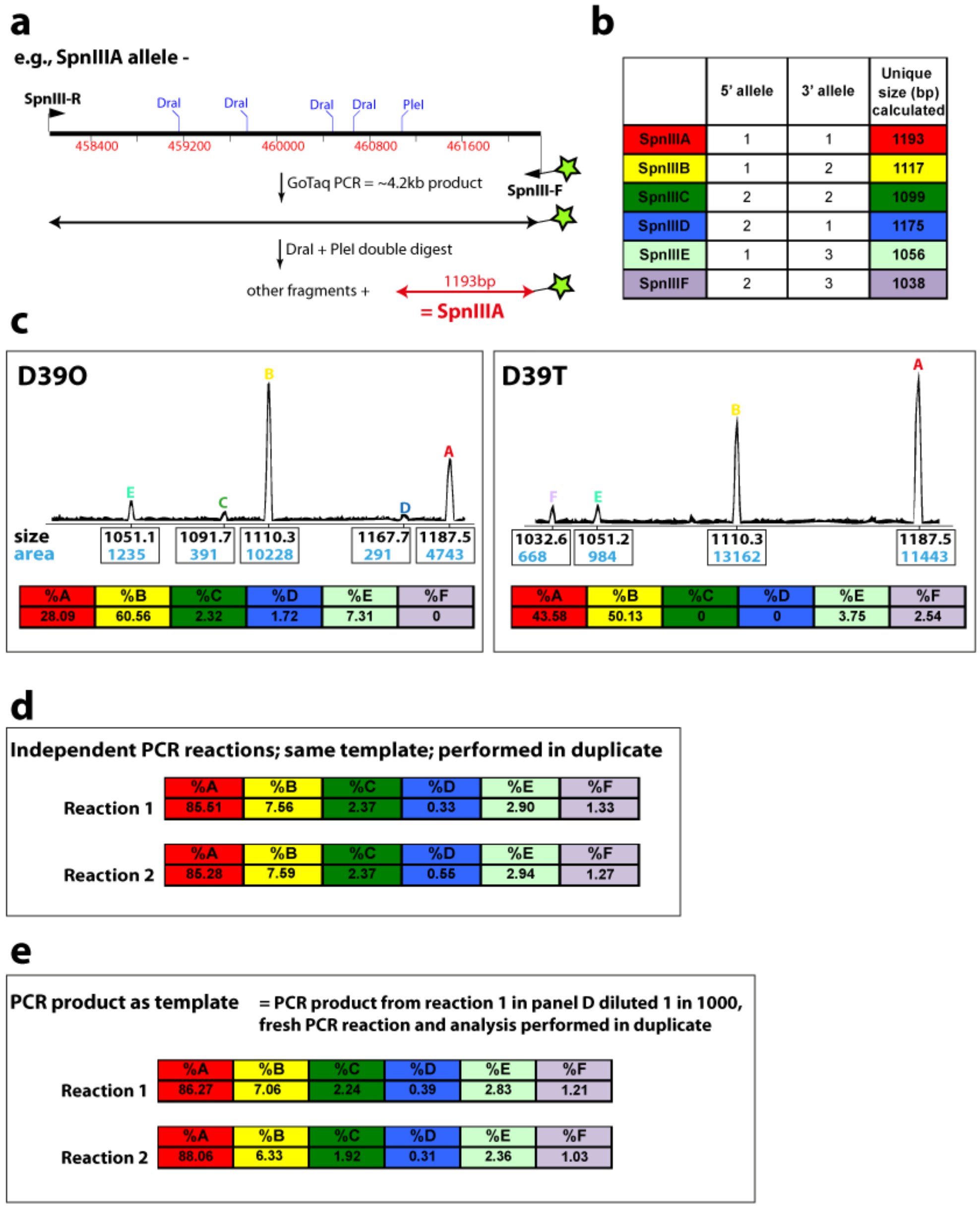

Supplementary Figure 6: Allele quantification. Illustration of the method used to characterise and quantify the six SpnD39III alleles using genomic DNA templates. Panel a shows an example of PCR using primer SpnD39III-F, containing a 6-FAM fluorescent label (green star) to allow gene scan analysis, and primer SpnD39III-R, at the SpnD39III locus of D39 genomic DNA. Following PCR and a double digest using DraI and PleI, a unique fragment size of 1193 bp is achieved. This would indicate the allele present at this particular locus is SpnD39IIIA. The 5' and 3' alleles that recombine to give the six SpnD39III alleles A-F at the SpnD39III locus, and the theoretical unique size of fluorescently labelled products generated by PCR and digestion for each of the six unique alleles, of each particular fluorescently labelled digestion product of the PCR products of the six alleles calculated *in silico*, is shown in (panel b). In all cases the actual size of all fragments (panel c) was 6bp smaller than the theoretical size calculated *in silico* (panel b). This is likely due to mapping fragments at the extreme upper limit of the LIZ1200 size standards (Life Technologies) required for quantification of these fluorescently labelled fragments. Panel c shows two examples of gene scan traces and resulting quantification of digested PCR products following fluorescently labelled PCR on samples of gDNA from opaque (D39OP) and transparent (D39TP) *S. pneumoniae* colonies. Panels d and e show analysis of reactions performed to verify accuracy, reproducibility and unbiased nature of the methodology used; Panel d shows the results of two independent PCR reactions performed using the same genomic DNA template, but performed using separate reagents on different days; Panel e shows the results of a PCR, digest and genescan analysis performed using the PCR product from reaction 1 in panel d; this PCR product was diluted 1 in 1000, and two replicate PCR reactions performed using 1 µl this diluted PCR product as template in a fresh PCR reaction, which was then digested and analysed as above.

	Taxonomy	Number of genomes with double *hsdS* modules*	Total number of genomes	Comment
Firmicutes	*Listeria monocytogenes*	12	60	uncommon
	Enterococcus faecalis	149	340	common
	Enterococcus faecium	7	232	rare
	Streptococcus pneumoniae	262	262	all isolates
	Streptococcus mitis	3	13	uncommon
	Streptococcus suis	8	21	common
	Streptococcus agalactiae	34	225	uncommon
Tenericutes	*Mycoplasma pulmonis*	1	1	common
Spirochaetes	*Treponema denticola*	16	16	all isolates
Bacteroidetes	*Bacteroides fragilis*	12	14	most isolates and in related species

Supplementary Table 1: Taxonomic distribution of inverted *hsdS* modules in bacterial pathogens. *NCBI nr database searched with BLASTP and accessed last in February 2014.

	Deviation			Significance of deviation (χ^2 test)			
Marker	Leading	Lagging	Both	Leading	Lagging	Total	Leading/Lagging
SpnD39IIIA	18%	112%	67%	0.01	7×10^{-63}	1×10^{-43}	5×10^{-14}
SpnD39IIIB	32%	71%	55%	1×10^{-7}	2×10^{-44}	6×10^{-45}	4×10^{-5}
SpnD39IIIC	-32%	-28%	-29%	1×10^{-8}	1×10^{-11}	1×10^{-18}	ns *
SpnD39IIID	-38%	-18%	-26%	7×10^{-9}	0.0010	6×10^{-10}	0.0052
SpnD39IIIE	37%	67%	54%	4×10^{-10}	3×10^{-39}	1×10^{-44}	0.0023
SpnD39IIIF	-9%	-14%	-12%	ns	0.0005	0.0002	ns
EcoKI	5%	2%	4%	ns	ns	ns	ns
EcoAI	-31%	-28%	-30%	4×10^{-8}	4×10^{-6}	7×10^{-13}	ns
EcoR124I	48%	50%	49%	6×10^{-31}	7×10^{-31}	4×10^{-60}	ns
EcoPI	-45%	-48%	-46%	8×10^{-196}	1×10^{-238}	$<10^{-300}$	0.066
EcoP15I	214%	198%	205%	1×10^{-300}	$<10^{-300}$	$<10^{-300}$	0.024

Supplementary Table 2: The deviation of the number of expected and observed target sites. By using nucleotide frequencies we can estimate the expected number of each target site using the assumption that nucleotides are distributed independently (i.e. the probability of finding a specific nucleotide in a specified position does not depend upon nucleotides in other positions). We tested the significance of the differences between expected and observed numbers for each marker site in both strands and in each strand separately. * ns non significant.

		Head-to-head		Tail-to-tail		Bidirectional	
Marker	Strand	R	p-value	R	p-value	R	p-value
SpnD39IIIA	leading	0.96	ns	0.94	ns	0.95	ns
	lagging	1.03	ns	1.09	0.0550	1.01	ns
SpnD39IIIB	leading	1.17	9×10^{-4}	1.21	0.0001	1.14	0.0092
	lagging	1.15	0.0002	1.17	1×10^{-5}	1.17	8×10^{-6}
SpnD39IIIC	leading	1.12	0.0770	1.04	ns	1.12	0.0913
	lagging	1.00	ns	1.09	0.0770	1.09	0.0509
SpnD39IIID	leading	0.91	ns	0.94	ns	0.88	ns
	lagging	1.07	ns	1.27	6×10^{-6}	1.11	0.0564
SpnD39IIIE	leading	1.02	ns	0.96	ns	0.94	ns
	lagging	1.05	ns	1.08	0.0342	1.07	0.0647
SpnD39IIIF	leading	1.09	ns	0.91	ns	1.02	ns
	lagging	0.96	ns	0.93	ns	0.99	ns
EcoAI	leading	0.89	ns	0.92	ns	0.89	ns
	lagging	0.94	ns	1.02	ns	1.00	ns
EcoKI	leading	1.01	ns	1.07	ns	1.14	0.0141
	lagging	0.96	ns	0.95	ns	0.95	ns
EcoR124I	leading	0.97	ns	1.01	ns	1.01	ns
	lagging	1.01	ns	1.09	0.0116	1.07	0.0333
EcoP15I	leading	1.42	$<10^{-300}$	1.45	$<10^{-300}$	1.53	$<10^{-300}$
	lagging	1.41	$<10^{-300}$	1.54	$<10^{-300}$	1.57	$<10^{-300}$
EcoPI	leading	1.24	$<10^{-300}$	1.18	$<10^{-300}$	1.21	$<10^{-300}$
	lagging	1.22	$<10^{-300}$	1.18	$<10^{-300}$	1.25	$<10^{-300}$

Supplementary Table 3: Clark-Evans test of degree of randomness of target site distribution. The distance to nearest neighbour distribution test or the Clark-Evans test is the test of degree of randomness of spatial distribution of objects. We developed a directed version of the Clark-Evans test and estimated the distribution of distances of the target sites in head-to-head and tail-to-tail positions. The null hypothesis of the test is that target sites are distributed independently and the P-value is the probability that the same or greater deviation from random target site distribution can be achieved by chance. * ns non significant.

Marker	Strand	*ADNN	p-value for head-to-head	p-value for tail-to-tail	p-value for bidirectional
SpnD39IIIA	leading	2,153	ns**	ns	ns
	lagging	4,210	ns	ns	ns
SpnD39IIIB	leading	1,557	ns	0.042	0.009
	lagging	2,765	ns	0.085	0.023
SpnD39IIIC	leading	2,407	ns	ns	ns
	lagging	4,780	ns	0.057	ns
SpnD39IIID	leading	3,602	ns	ns	ns
	lagging	7,204	ns	ns	ns
SpnD39IIIE	leading	1,598	ns	0.003	0.098
	lagging	2,650	ns	0.018	ns
SpnD39IIIF	leading	2,021	0.014	ns	ns
	lagging	3,552	0.097	ns	ns
EcoAI	leading	12,025	ns	ns	ns
	lagging	11,157	ns	ns	ns
EcoKI	leading	7,893	ns	ns	ns
	lagging	7,761	ns	ns	ns
EcoR124I	leading	2,865	ns	ns	ns
	lagging	2,667	ns	ns	ns
EcoP15I	leading	663	0.0007	0.093	0.0001
	lagging	638	0.001	ns	0.0005
EcoPI	leading	928	0.042	ns	ns
	lagging	953	0.093	ns	ns

Supplementary Table 4: Kolmogorov-Smirnov test for distribution pairs of target sites at low distance. We estimated the number of pairs of target sites with distances which are less than or equal to a specified distance for randomly distributed markers, in addition we calculated the observed number of such pairs. We considered the short distance effect and analysed the distribution of pairs of target site with distances less than or equal to the *Average Distance to Nearest Neighbour (ADNN). This empirical distribution was compared with the distribution of distances for the randomly and uniformly distributed target sites using a Kolmogorov-Smirnov test. For the directed case we considered the pairs in head-to-head, tail-to-tail and bidirectional positions. ** ns non significant.

	SpnD39IIIA		SpnD39IIIB		SpnD39IIIC		SpnD39IIID		SpnD39IIIE		SpnD39IIIF	
	Nasal wash	Colony	Nasal wash	Colony	Nasal wash	Colony	Nasal wash	Colony	Nasal wash	Colony	Nasal wash	Colony
Day1, Sample1	3.8	9.3	12.4	5.7	9.8	0	1.6	1.1	71.5	83.4	0.9	0.5
Day1, Sample2	5.4	9.1	15.3	11.0	0	0	1.2	1.2	77.2	78	0.9	0.7
Day1, Sample3	11.5	10.3	0	5.6	0	0	0.5	1.8	86.7	81.8	1.3	0.5
Day1, Sample4	12.1	8.2	5.67	16.9	0	0	1.1	1.2	79.56	73.4	1.6	1.1
Day1, Sample5	9.3	9.3	10.8	13.0	0	0	0.8	1.7	78.9	75.5	0.1	0.4
Day3, Sample1	7.4	8.6	5.2	16.4	0.4	0	0.9	0.9	85.7	73.8	0.3	0.3
Day3, Sample2	5.9	6.5	13.5	20.3	0	0	0.8	0.7	79.7	72.3	0	0.3
Day3, Sample3	4.6	7.9	11.7	8	0	0.8	1.1	8.9	82.7	73.3	0	1.1
Day3, Sample4	26.2	23.5	5.6	7.1	0	0.4	1.3	3.2	66.7	64.4	0.3	1.4
Day3, Sample5	9	8.8	2.8	7.2	0.4	0	1.1	2.3	86.4	81.3	0.3	0.4
Day7, Sample1	27.2	36.6	9.3	4.0	0	0	0.8	0.9	62.3	57.5	0.5	0.9
Day7, Sample2	7.0	13.8	6.2	8.1	0	0	0.9	2.1	85.8	75.7	0	0.4
Day7, Sample3	9.3	10.2	1.1	4.9	0	0	1.2	1.4	88.4	83.1	0	0.5
Day7, Sample4	6.3	9.8	6	7.4	0	0	1	1.5	86.8	80.8	0	0.5
Day7, Sample5	8.1	8.5	21.4	6.9	0.3	0	1.5	1.6	65.2	82.1	3.5	0.8
Inoculum replica 1	11l0	9.4	8.5	11.3	0	0.4	1.4	1.2	78.6	77.1	0.5	0.6
Inoculum replica 2	10.0	8.7	8.4	10.7	0	0	1.1	1.3	80.0	78.8	0.5	0.5
Inoculum replica 3	9.4	9.0	8.0	9.8	0	0	1.2	1.3	80.8	79.4	0.6	0.6

Supplementary Table 5: **Comparison of SpnD39III allele quantification (%) in nasal wash samples and first passage bacterial colonies**. Nasal wash refers to DNA extracted directly from 100µl of un-plated nasal lavage (reported in Supplementary Fig. 5), colonies refers to DNA extracted from a pool of approximately 100 colonies grown on TSA + 3% blood of the same nasal wash sample.

Supplementary References

1 Flusberg, B.A. et al., "Direct detection of DNA methylation during single-molecule, real-time sequencing," Nat. Methods 7(6), 461 (2010).
